# The Prevalence of Insufficient Sleep and Bedtime Delay Among Kindergarten Children Aged 3 to 6 Years in a Rural Area of Shanghai: A Cross-Sectional Study

**DOI:** 10.3389/fped.2021.759318

**Published:** 2021-11-25

**Authors:** Xi Chen, Yan Qiang, Xia Liu, Qing Yang, Qingqing Zhu, Bin Li, Ruiping Wang

**Affiliations:** ^1^Shanghai Skin Disease Hospital, Shanghai, China; ^2^School of Public Health, Shanghai University of Traditional Chinese Medicine, Shanghai, China; ^3^Songjiang Maternity and Child Health Hospital, Shanghai, China

**Keywords:** kindergarten children, insufficient sleep, bedtime delay, sleep deprivation, prevalence

## Abstract

**Introduction:** Sleep deprivation in children is a global public health problem that affects the physical and mental development of children. Bedtime delay induced by longer screen time and heavy study load is a common cause of sleep deprivation in children. However, the prevalence of insufficient sleep and bedtime delay and related influencing factors among kindergarten children is limited in Shanghai, China.

**Methods:** In 2018, we selected 8,586 children aged 3 to 6 years and their parents in Songjiang District, Shanghai. Data was collected among parents by face-to-face questionnaire interview with signed informed consent. We defined insufficient sleep as those who slept for < 10 h in children aged 3–5 years or 9 h in children aged 6 years within 24 h, and we define bedtime delay as children with bedtime after 21:00. SAS 9.1.3 software was used to calculate the prevalence of bedtime delay and insufficient sleep, and logistic regression was used to explore potential influencing factors.

**Results:** The prevalence of insufficient sleep and bedtime delay among children aged 3 to 6 years was 11.67 and 56.85%, respectively. The prevalence of insufficient sleep among boys was slightly higher than among girls [odds ratio (OR) = 1.18, 95% confidence interval (CI): 1.04–1.35]. With the increase of the age of children, the prevalence of insufficient sleep increased gradually (*P* < 0.05). The prevalence of insufficient sleep was higher among the only child in the family (OR = 1.18, 95% CI: 1.02–1.36) and those with longer hours of TV watching (OR = 109, 95% CI: 1.02–1.16). Meanwhile, the prevalence of bedtime delay was also higher among the only child in the family (OR = 1.17, 95% CI: 1.06–1.79), among those with parents accompanying for sleep (OR = 1.21, 95% CI: 1.10-1.34), and those with longer TV watching time (OR = 1.13, 95% CI: 1.07–1.18).

**Conclusions:** Insufficient sleep and bedtime delay were prevalent in Chinese children aged 3 to 6 years, especially in boys and older children. TV watching as well as parents accompanying for sleep were associated with insufficient sleep and bedtime delay. We recommend that parents should limit the screen time of children, advocate earlier bedtime and later morning wake-up among children, as well as make their children sleep in separate beds or rooms in younger age.

## Introduction

Sleep is essential for optimal health in children. Healthy sleep requires adequate duration, appropriate timing, good quality, regularity, and without sleep disturbance or disorders (1, 2). Insufficient sleep is associated with attention, behavior, and learning problems and increases the risk of injuries, obesity, diabetes, and depression, which is a public health problem globally (3–6). Sleep deprivation has been recognized as one of the most common sleep problems encountered by general practitioners (7). In children, sleep deprivation has been paid much more attention due to the essential stage of physical and mental development. Previous research indicated that the prevalence of insufficient sleep in children displayed an upward tendency with increasing age, and about 10% of children aged 1 to 3 years have sleep problems (8), while 15–30% of preschoolers have insufficient sleep (9). In adolescents aged 8 to 13 years, 71% of them were identified as short sleepers (10). In the United States, the prevalence of insufficient sleep among middle school students was 57.8% in the year 2015 and 72.7% among high school students (11). Increasing evidence demonstrated that sleep deprivation in children was closely related to their physical and mental development, such as language, memory, attention, behavior, cognition, emotion, etc. (12), which affects children and their families in short term and long term as well.

Sleep time in children is affected by many internal and external influencing factors. The sleep condition in children can be regulated by the hypothalamic–pituitary–adrenal cortex and the sympathetic adrenal–marrow system, brain structure, and mutation of the human gene, such as transcriptional repressor gene hDEC2-P385R, and chronotype (13–16). However, insufficient sleep in healthy children is more likely to be influenced by external factors, including sociodemographic factors, such as the age of children, sex, socioeconomic status, education level of the parents, family income, social jetlag, earlier school start times, etc. (2, 17). In addition, the lifestyle of children is another external influencing factors for insufficient sleep, such as pre-sleep TV watching and bedtime delay (18). Less TV watching time and a regular and earlier bedtime can help children get adequate sleep and improve their sleep quality (19).

In China, previous research demonstrate that sleep problem, such as insufficient sleep among children, is very common. One research published in 2016 showed that the prevalence of too long or too short sleep time among children aged 3 to 6 years in urban China was 15.1% (2). In recent years, with the rapid economic development and the process of urbanization, the Chinese government paid more attention on the quality of education among children. The Chinese Ministry of Education (CME) promulgated regulations to alleviate the academic burden of children for the purpose of all-round development of children. With the implementation of the CME regulations, children had more spare time for hobby development and had more estimated rest time, which was supposed to lower the sleep problem in children. Although there was evidence of the prevalence and related influencing factors of insufficient sleep and sleep delay among children from urban areas in China, the data was still limited in rural areas of China. In this study, we investigated the prevalence of insufficient sleep and bedtime delay among children aged 3 to 6 years in Songjiang District of Shanghai to explore the potential influencing factors of insufficient sleep and bedtime delay so as to provide basic data for future intervention strategy development.

## Methods

### Study Population

We conducted this study during March and September in 2018 in Songjiang District of Shanghai, China. PASS software was applied for sample size calculation, we assumed that the prevalence of insufficient sleep was 10% in Songjiang District, considering an inspection level (α) of 0.05 a permissible error (δ) of 0.007 (7% of insufficient sleep prevalence) and design effect of 1.2 due to non-simple random sampling strategy; at least 8,467 children should be sampled and recruited. Taking the population size of kindergarten into consideration, we finally selected 20 kindergartens in this study in Songjiang District of Shanghai. We applied the cluster sampling method to select the study population. First, we randomly selected 20 kindergartens out of all 115 kindergartens in Songjiang District. Second, we recruited all children aged 3 to 6 years and their parents from the 20 selected kindergartens. Overall, a total number of 8,626 children and their parents were recruited in this study. Finally, a total of 8,586 questionnaires were completed by face-to-face interview and were included in the final analysis. The Songjiang Maternal and Child Health-care Hospital Institution Review Board (SSDH-IEC-SG-057-3.1) gave the ethic approval of this study on December 2017 (IRB#20171203). The research coordinators orally communicated with each child and their parents and then had them sign the informed consent papers ahead of the questionnaire interview.

### Data Collection

Data were collected by using a questionnaire which was designed by a specific research team from Songjiang Maternal and Children's Health-care Hospital and which was administered by trained interviewers. In this study, questionnaire for data collection included three parts. Part one included 10 demographic questions (age and sex of children, age and education of parents, family yearly income, ethnicity, residency status, and only-child status of their family). Part two included 12 questions of ordinary sleep habits and sleep problems (e.g., “when will your child go to bed in the night?,” “when will your child fall asleep?,” “when will your child wake-up in the morning?,” “how many hours does your child sleep in the daytime?,” “is your child accompanied by parents when they sleep in the night?,” “does your child watch TV before sleep?,” “does your child have nightmares randomly?,” etc.). Part three included information for follow-up contact of parents and their children. The details of the questionnaire are shown in the Supplementary Material. A pilot study demonstrated that the split-half reliability coefficient of the questionnaire was 0.83, and the content validity coefficient was 0.82. All the participants were interviewed face to face, in Chinese, after signing the informed consent.

### Definition and Index Calculation

In this study, we defined “only child” as a child who has no brothers or sisters in their family. According to the consensus of the American Academy of Sleep Medicine, insufficient sleep among children was defined as those who slept for < 10 h with age of 3 to 5 years and < 9 h for those with age of 6 years, within each 24 h (including naps) (20). Children with bedtime after 21:00 were defined as cases of bedtime delay in this study (21). We calculated the prevalence of insufficient sleep as the number of children with insufficient sleep divided by the total number of children and the prevalence of bedtime delay as the number of children with bedtime after 21:00 divided by the total number of children. In this study, we recorded the education of parents as having completed schooling years and classified it into four categories, including school of junior high or lower (0–9 years), school of senior high (10–12 years), college (13–16 years), and postgraduate and above (>16 years). Family yearly income (CNY) was categorized into five categories (<50,000, 50,000–10,000, 100,001–150,000, 150,001–300,000, and >300,000).

### Data Analysis

SAS software (version 9.1.3) was applied for statistical analysis. In this study, data was described as means as well as standard deviations or median as well as interquartile range for quantitative variables and frequency and/or prevalence for qualitative variables. Student's *t*-test or Wilcoxon rank–sum test was used to examine the difference in the age of father and age of mother and to examine the difference in TV watching time between children with and without sufficient sleep as well as children with and without bedtime delay. Chi-square test was applied to examine the difference of bedtime delay prevalence and insufficient sleep prevalence among children with different demographic characteristics. Multi-variable logistic regression was used to calculate the odds ratio (OR) and 95% confidence interval (95% CI) of insufficient sleep prevalence and bedtime delay prevalence between kindergarten children with different demographic features so as to explore the influencing factors of insufficient sleep and bedtime delay among children aged 3–6 years. Figures were produced to depict the detailed information of daytime sleep, night sleep, and morning wake-up among children of different sex and ages. In this study, *P*-values < 0.05 were considered statistically significant.

## Results

In this study, 8,586 kindergarten children and their parents were finally analyzed. The 8,586 children included 4,595 boys (53.52%), and the proportion of children aged 3, 4, 5, and 6 years was 17.76, 34.13, 32.05, and 16.06%, respectively. The average age of mothers and fathers was 32.17 ± 3.96 years old and 34.04 ± 4.82 years old, respectively. Meanwhile, the majority of fathers and mothers had college education; the proportion was 63.69 and 65.55% for each. Furthermore, 43.68% of children had a family income of over 300,000 CNY per year, 52.94% of children were local residents, and 62.44% of them were the only child of their family. Table 1 demonstrates that the proportion of “only child in the family” among boys (63.90%) was higher than among girls (60.76%); the difference was statistically significant (*P* < 0.05; Table 1).

**Table 1:** The demographic features of children aged 3–6 years and their parents in a rural area of Shanghai, China, in 2018 (*n* = 8,586).

**Variables**	**Total condition (*n* = 8,586)**	**Sex of children**		***X^**2**^/t***	***P-*value**
		**Boys (*n* = 4,595)**	**Girls (*n* = 3,991)**		
**Age of children (years)**, ***n*** **(%)**				0.801	0.847
3	1,525 (17.76)	820 (17.85)	705 (17.66)		
4	2,930 (34.13)	1,570 (34.17)	1,360 (34.08)		
5	2,752 (32.05)	1,456 (31.69)	1,296 (32.47)		
6	1,379 (16.06)	749 (16.30)	630 (15.79)		
Age of mother (years), mean (SD)	32.17 (3.96)	32.15 (3.95)	32.19 (3.98)	0.470	0.641
Age of father (years), mean (SD)	34.04 (4.82)	33.99 (4.78)	34.09 (4.86)	0.900	0.366
**Education of mother**, ***n*** **(%)**				3.079	0.380
Junior high or under	866 (10.09)	472 (10.27)	394 (9.87)		
Senior high	1,613 (18.79)	877 (19.09)	736 (18.44)		
College	5,628 (65.55)	3,006 (65.42)	2,622 (65.70)		
Postgraduate and above	479 (5.58)	240 (5.22)	239 (5.99)		
**Education of father**, ***n*** **(%)**				3.724	0.293
Junior high or under	684 (7.97)	390 (8.49)	294 (7.37)		
Senior high	1,705 (19.86)	910 (19.80)	795 (19.92)		
College	5,468 (63.69)	2,910 (63.33)	2,558 (64.09)		
Postgraduate and above	729 (8.49)	385 (8.38)	344 (8.62)		
**Family yearly income (CNY)**, ***n*** **(%)**				3.978	0.409
<50, 000	1,344 (15.65)	730 (15.89)	614 (15.38)		
50,000–100,000	1,206 (14.05)	616 (13.41)	590 (14.78)		
100,001–150,000	1,117 (13.01)	601 (13.08)	516 (12.93)		
150,001–300,000	1,169 (13.62)	640 (13.93)	529 (13.25)		
Over 300,000	3,750 (43.68)	2,008 (43.70)	1,742 (43.65)		
**Ethnics**, ***n*** **(%)**				0.229	0.633
Han	8,348 (97.23)	4,464 (97.15)	3,884 (97.32)		
Minority	238 (2.77)	131 (2.85)	107 (2.68)		
**Residency status**, ***n*** **(%)**				3.533	0.060
Local resident	4,545 (52.94)	2,389 (51.99)	2,156 (54.02)		
Non-local resident	4,041 (47.06)	2,206 (48.01)	1,835 (45.98)		
**The only child in the family**, ***n*** **(%)^a^**				8.943	0.003
Yes	5,361 (62.44)	2,936 (63.90)	2,425 (60.76)		
No	3,225 (37.56)	1,659 (36.10)	1,566 (39.24)		

a *The differences between groups was statistically significant (P < 0.05)*.

### The Bedtime, Wake-Up Time, and Sleep Time Among Kindergarten Children

**Figure 1:**
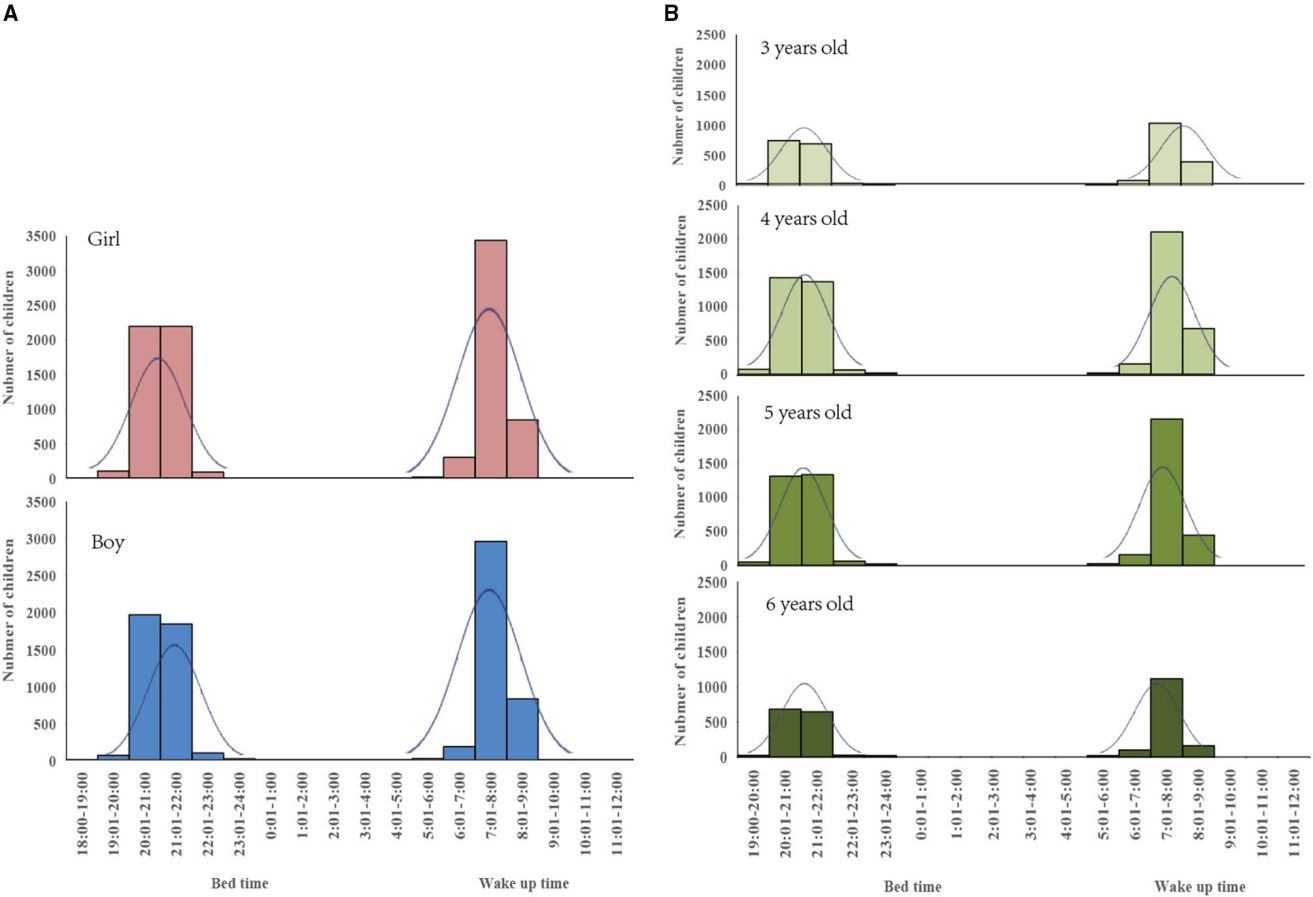
The frequency distribution of the bedtime and wake-up time of children between boys and girls and between children aged 3, 4, 5, and 6 years in the year 2018 in Shanghai, China. **(A)** Bedtime and Wake up time for children with different sex. **(B)** Bedtime and Wake up time for children with different age.

Figure 1 indicates that the majority of children went to bed between 20:00 and 22:00 at night and woke up between 7:00 and 8:00 in the morning, both for girls and boys as well as among children aged 3, 4, 5, and 6 years. The average bedtime of boys was slightly later than that of girls, while the average wake-up time was comparable between boys and girls, so the total sleep time among boys was slightly shorter than girls (Figure 1). The average bedtime was similar among children aged 3–6 years, but the average wake-up time was earlier among children aged 6 years than children aged 3 or 4 years, so the total sleep time among children aged 6 years was shorter than that of children aged 3 or 4 years (Figure 1).

**Figure 2:**
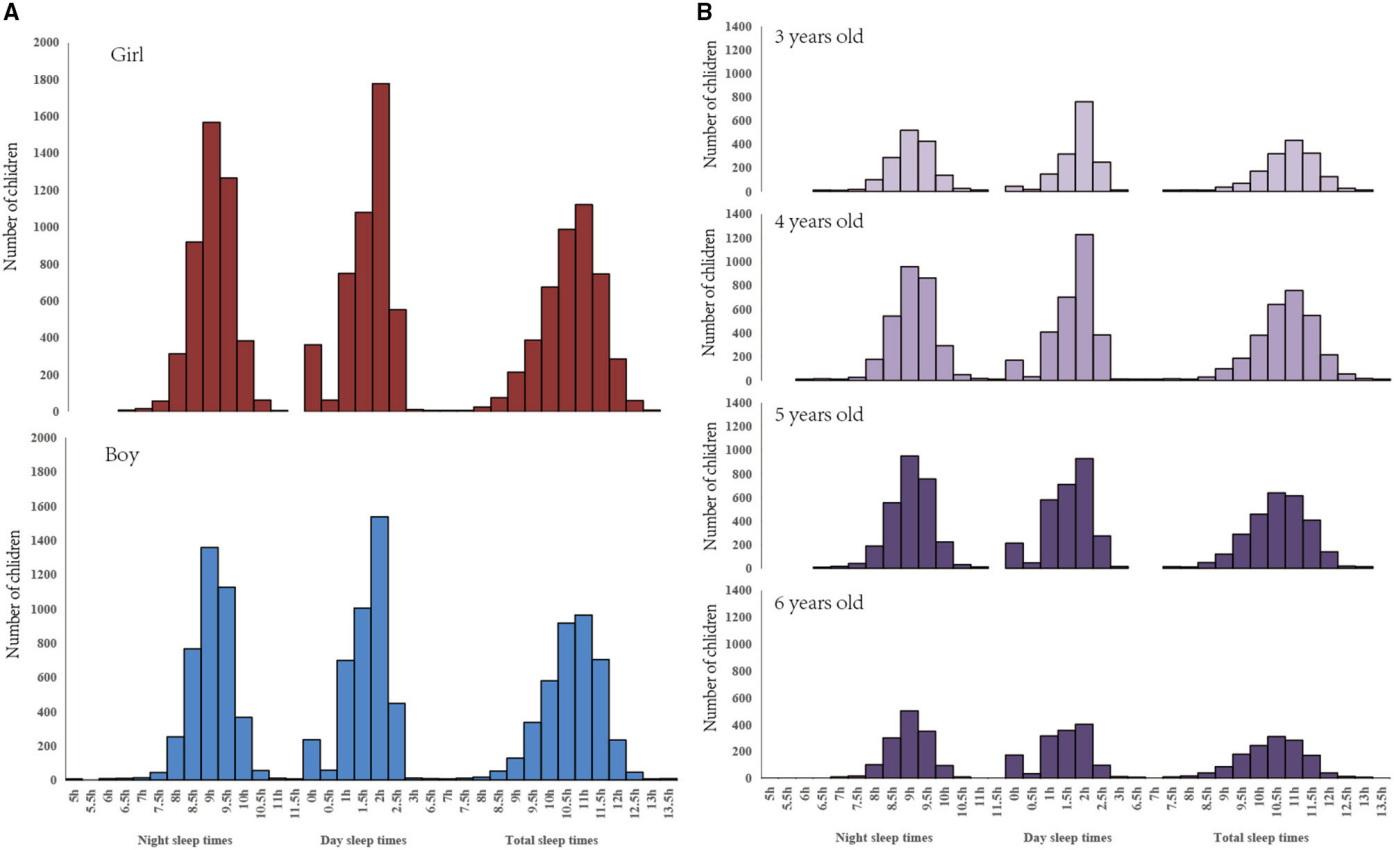
The frequency distribution of the sleep duration of children in the daytime, night, and whole day between boys and girls and between children aged 3, 4, 5, and 6 years in the year 2018 in Shanghai, China. **(A)** Sleep times among children with different sex. **(B)** Sleep times among children with different ages.

Figure 2 indicates that the majority of night sleep time, daytime sleep time, and total sleep time was 9, 2, and 11 h, respectively, both for boys and girls, but the average of total sleep time among girls was slightly longer than among boys (Figure 2). The majority of night sleep time was 9 h and of daytime sleep time was 2 h, among children aged 3–6 years, but for total sleep time, most of the children aged 3 and 4 years slept for 11 h, and most of the children aged 5 and 6 slept for 10.5 h; the younger children slept longer than the older children (Figure 2).

### The Prevalence of Insufficient Sleep and Bedtime Delay Among Children

In this study, the prevalence of insufficient sleep among children aged 3 to 6 years was 11.67% (1,785/8,586). Table 2 indicates that the prevalence of insufficient sleep in boys (12.51%) was higher than that in girls (10.70%) (*P* < 0.05). With the increase of the age of children, the prevalence of insufficient sleep increased gradually (*P* < 0.05). Children who were the only child in the family had a statistically significant higher prevalence of insufficient sleep (12.12%) than children who were not (10.91%). Moreover, compared with children with sufficient sleep, children with insufficient sleep spent more time on TV watching (*P* < 0.05). However, the age of parents and education of parents had no impact on the prevalence of insufficient sleep in children, and there was no significant difference in insufficient sleep prevalence among children with different ethnicity, different residency status, and parents accompanying for sleep (*P* > 0.05; Table 2).

**Table 2:** The prevalence of bedtime delay and insufficient sleep among children aged 3–6 years in a rural area of Shanghai, China, in the year 2018.

**Variables**	**Insufficient sleep**		**Bedtime delay**	
	**Yes (*n* = 1,002)**	**No (*n* = 7,584)**	**Yes (*n* = 4,881)**	**No (*n* = 3,705)**
**Sex**, ***n*** **(%)^a^**				
Boys	575 (12.51)	4,020 (87.49)	2,623 (57.08)	1,972 (42.92)
Girls	427 (10.70)	3,564 (89.30)	2,258 (56.58)	1,733 (43.42)
**Age of children (years)**, ***n*** **(%)^a^**				
3	96 (6.30)	1,429 (93.70)	864 (56.66)	661 (43.34)
4	265 (9.04)	2,665 (90.96)	1,638 (55.90)	1,292 (44.10)
5	375 (13.63)	2,377 (86.37)	1,591 (57.81)	1,161 (42.19)
6	266 (19.29)	1,113 (80.71)	788 (57.14)	591 (42.86)
Age of mother (years), mean (SD)^b^	32.29 (4.08)	32.16 (3.94)	32.29 (3.97)	32.02 (3.95)
Age of father (years), mean (SD)^b^	34.23 (4.65)	34.01 (4.84)	34.12 (4.82)	33.93 (4.82)
**Education of mother**, ***n*** **(%)**				
Junior high or under	98 (11.32)	768 (88.68)	494 (57.04)	372 (42.96)
Senior high	168 (10.42)	1,445 (89.58)	878 (54.43)	735 (45.57)
College	674 (11.98)	4,954 (88.02)	3,230 (57.39)	2,398 (42.61)
Postgraduate and above	62 (12.94)	417 (87.06)	279 (58.25)	200 (41.75)
**Education of father**, ***n*** **(%)**				
Junior high or under	81 (11.84)	603 (88.16)	393 (57.46)	291 (42.54)
Senior high	188 (11.03)	1,517 (88.97)	938 (55.01)	767 (44.99)
College	653 (11.94)	4,815 (88.06)	3,110 (56.88)	2,358 (43.12)
Postgraduate and above	80 (10.97)	649 (89.03)	440 (60.36)	289 (39.64)
**Ethnics**, ***n*** **(%)**^**b**^				
Han	977 (11.70)	7,371 (88.30)	4,360 (57.02)	3,588 (42.98)
Minority	25 (10.50)	213 (89.50)	121 (50.84)	117 (49.16)
**Residency status**, ***n*** **(%)**^**b**^				
Local resident	541 (11.90)	4,004 (88.10)	2,531 (55.69)	2,014 (44.31)
Non-local resident	461 (11.41)	3,580 (88.59)	2,350 (58.15)	1,691 (41.85)
**Parents accompany for sleep**, ***n*** **(%)**^**b**^				
Yes	718 (11.61)	5,465 (88.39)	3,610 (58.24)	2,582 (41.76)
No	284 (11.82)	2,119 (88.18)	1,280 (53.27)	1,123 (46.73)
**The only child in the family**, ***n*** **(%)**^**a, b**^				
Yes	650 (12.12)	4,711 (87.88)	3,097 (57.77)	2,264 (42.23)
No	352 (10.91)	2,873 (89.09)	1,784 (55.32)	1,441 (44.68)
Time of TV watching (h), mean (SD)^a^^,^^b^	1.54 (1.09)	1.48 (0.92)	1.52 (0.97)	1.43 (0.92)

a *The differences in prevalence of insufficient sleep between groups was statistically significant (P < 0.05)*.

b *The differences in prevalence of bedtime delay between groups was statistically significant (P < 0.05)*.

The prevalence of bedtime delay among children aged 3 to 6 years was 56.85%, which was 57.08% for boys and 56.58% for girls. In comparison with children without bedtime delay, the ages of the mothers and fathers of children with bedtime delay were both slightly older (*P* < 0.05). The prevalence of bedtime delay was significantly higher in children with Han ethnicity than in children with minority ethnicity, and the prevalence was significant lower in local resident children than non-local resident children (*P* < 0.05). A higher prevalence of bedtime delay was identified among children with parents accompanying for sleep than those children without (*P* < 0.05). Children who were the only child in the family had a higher prevalence of bedtime delay (57.77%) than those who were not the only child in the family (55.32%) (*P* < 0.05), and children with bedtime delay had a longer time of TV watching than children without bedtime delay (*P* < 0.05; Table 2).

### The Influencing Factors of Insufficient Sleep and Bedtime Delay Among Children

In this study, the prevalence of insufficient sleep among boys was slightly higher than among girls (OR = 1.18, 95% CI: 1.04–1.35). In comparison with children aged 3 years, the prevalence of insufficient sleep was higher among children aged 4 years (OR = 1.48, 95% CI: 1.16–1.89), 5 years OR = 2.38, 95% CI: 1.88–3.00), and 6 years (OR = 3.61, 95% CI: 2.82–4.62). The prevalence of insufficient sleep was higher among children who were the only child in the family (OR = 1.18, 95% CI: 1.02–1.36) and those with longer hours of TV watching (OR = 1.09, 95% CI: 1.02–1.16; Table 3).

**Table 3:** The influencing factors for bedtime delay and insufficient sleep among children aged 3–6 years in a rural area of Shanghai, China, in the year 2018.

**Variables**	**Insufficient sleep (LRa)**		**Bedtime delay (LRb)**	
	**OR**	**95% CI**	**OR**	**95% CI**
**Sex**, ***n*** **(%)**				
Boys	1.18	1.04–1.35	1.01	0.92–1.10
Girls	1.00	-	1.00	-
**Age of children (years)**, ***n*** **(%)**				
3	1.00	-	1.00	-
4	1.48	1.16–1.89	0.97	0.85–1.09
5	2.38	1.88–3.00	1.06	0.93–1.20
6	3.61	2.82–4.62	1.04	0.89–1.21
**The only child in the family**, ***n*** **(%)**				
Yes	1.18	1.02–1.36	1.17	1.07–1.28
No	1.00		1.00	-
**Ethnics**, ***n*** **(%)**				
Han	-	-	1.38	1.06–1.79
Minority	-	-	1.00	-
**Residency status**, ***n*** **(%)**				
Local resident	-	-	0.85	0.78–0.93
Non-local resident	-	-	1.00	-
**Parents accompany for sleep**, ***n*** **(%)**				
Yes	-	-	1.21	1.10–1.34
No	-	-	1.00	-
Age of mother (years), mean (SD)	-	-	1.02	1.01–1.04
Age of father (years), mean (SD)	-	-	1.00	0.99–1.01
Time of TV watching (h), mean (SD)	1.09	1.02–1.16	1.13	1.07–1.18

*LRa, multi-variate logistic regression to explore influencing factors for insufficient sleep among kindergarten children aged 3–6 years; LRb, multi-variate logistic regression to explore influencing factors for bedtime delay among kindergarten children aged 3–6 years*.

Table 3 indicates that the prevalence of bedtime delay was higher among children who were the only child in the family (OR = 1.17, 95% CI: 1.06–1.79) and higher among children with Han ethnicity (OR = 1.38, 95% CI: 1.06–1.79). Children with parents accompanying for sleep had a higher prevalence of bedtime delay (OR = 1.21, 95% CI: 1.10–1.34), and children with longer TV watching time also had a higher prevalence of bedtime delay (OR = 1.13, 95% CI: 1.07–1.18; Table 3).

## Discussion

This cross-sectional study examined the prevalence and influencing factors of insufficient sleep and bedtime delay among children aged 3 to 6 years in a rural area of Shanghai, China. The results indicated the following important trends: (1) the prevalence of insufficient sleep and bedtime delay among children aged 3 to 6 years was 11.67 and 56.85%, respectively; (2) sex, age of children, being the only child in the family, and time of TV watching were influencing factors for insufficient sleep of children, and being the only child in the family, ethnicity, residency status, time of TV watching, and parents accompanying for sleep were influencing factors for the bedtime delay of children; and (3) the higher prevalence of insufficient sleep among boys might be attributed to the later bedtime at night, and the higher prevalence of insufficient sleep among older children than among younger children might be attributed to the earlier wake-up time in the morning.

Previous studies showed that the prevalence of sleeping too long or too short among Chinese urban children aged 3 to 6 years old was 15.1% (2). In Australia, 10.9% of indigenous children do not get enough sleep (22). The prevalence of insufficient sleep in Netherlands, United Kingdom, and the United States was much higher, which was up to 25% (23). In this study, the prevalence of insufficient sleep among children aged 3 to 6 years in the Songjiang District of Shanghai was 11.67%, which was comparable to the prevalence of insufficient sleep in previous studies (11). The prevalence of insufficient sleep in this study was slightly higher than that in Australian children but lower than that in European children. This might be due to the different lifestyle and different education and sleep deprivation management between these countries. In China, the majority of parents would make a strict plan for bedtime of their children, especially among kindergarten children, and most of them would usually urge their children to go to bed before 22:00. This might contribute to the relatively lower prevalence of insufficient sleep in Chinese children. Previous studies demonstrated that the prevalence of bedtime delay among Chinese urban children aged 3–6 years was 69.5% (2). In this study, the prevalence of sleep delay among children in the rural areas of Shanghai was lower than that in urban areas, which may be related to regional economic development. Consistent with the matter of the lower prevalence of insufficient sleep in Australia, the prevalence of bedtime delay in indigenous Australian children (50%) is also lower than that in Chinese children (56.85%) (22). Therefore, taking measures to encourage kindergarten children to go to bed earlier is crucial for reducing the prevalence of insufficient sleep and bedtime delay.

This study indicated that the sex of children is one of the influencing factors for insufficient sleep. A previous cohort study showed that girls aged 3 to 6 years old slept 5 to 10 min longer than boys at all ages in the United Kingdom. In comparison with boys, the longer sleep time among girls was attributed to their later wake-up time in the morning (24). In line with the results of the above-mentioned previous study, this study indicated that the prevalence of insufficient sleep in girls was lower than that in boys, whereas the longer sleep time among girls in this study was attributed to their earlier bedtime at night. The reasons for the later bedtime in boys may be due to the fact that boys are more physically active and spend more screen time before bedtime than girls (25, 26). Besides this, there are more boys in one-child families in China due to the fact that the majority of parents who gave birth to a boy child first would not like to have a second baby. This might be a reason to explain why the only child had a higher prevalence of insufficient sleep in this study.

Previous studies showed that the age of children is another influencing factor for insufficient sleep in children (24, 26). A study of the sleep data of 690,747 children, which covered 20 countries, showed that the sleep duration of children aged 5 to 18 continued to decline rapidly (27). Consistent with previous studies, this study demonstrated that the total sleep time decreased, and the prevalence of insufficient sleep increased with the increase of age among 3- to 6 year old children. The further analysis suggesting that the older children slept shorter than younger children was attributed to the earlier wake-up time in the morning. The possible reason for 6 year old children waking up earlier than younger children was due to the starting time of kindergarten depending on the age of the children. For 6 year old children, their starting time was about 30 min earlier than that of the younger children. Another possible explanation was that the needed sleep length for 6 year old children was 1 h less than that of younger children; thus, the earlier rise times of 6 year old children might reflect the diminution of the needed sleep length. Thus, Chinese parents advocating to ensure that older children, who were awakened by their parents or by an alarm clock, delay their morning wake-up time and ensure early-night bedtime would be helpful for children with problems of insufficient sleep as well as bedtime delay.

Increasing number of studies reported that screen time was negatively associated with sleep time, especially the night screen time (28–31). This study showed that insufficient sleep and bedtime delay were related to a longer time of TV watching, which was consistent with previous studies. The American Association of Pediatric recommended that children aged 2 years and older should spend no more than 1 h a day in front of the screen, so parents need to monitor the screen exposure of their children, which can increase the sleep time (32). In this study, children with insufficient sleep or bedtime delay spent about 1.5 h each day on TV watching. Therefore, we recommended that parents should strengthen the control of TV watching time among children to < 1 h.

It has been accepted that children who were accompanied by their parents for sleep were more vulnerable to sleep deprivation (33). In this study, the scenario of parents accompanying for sleep was associated with bedtime delay among children but not associated with insufficient sleep among children. Moreover, the higher proportion of children who were the only child in the family in China predicted a high demand of parents accompanying for sleep, which was an important sleep problem in China, so we recommended that sleeping in separate beds or rooms earlier for children was helpful to deal with the bedtime delay problem and insufficient sleep as well.

In summary, regarding the large sample number of 8,586 children, which accounts for about 17% of children aged 3–6 years in kindergartens in Songjiang District, in this investigation, the final sample can be considered as a good representative of the population in Songjiang District. In this study, statistical analysis showed that the influencing factors of insufficient sleep were sex, age, being the only child in the family, and TV watching time, and the influencing factors of bedtime delay includes being the only child in the family, ethnicity, parents accompanying for sleep, and TV watching time. Due to the large sample size, some differences and associations with statistical significance seem to have little practice relevance. Taking practice relevance into consideration, the most relevant factors for insufficient sleep and bedtime delay were sex, age of children, and parents accompanying for sleep.

This study has some limitations. First, the study design of a cross-sectional study may induce some information bias and only allow the calculation of prevalence. Second, the self-reported information of bedtime at night, morning wake-up time, as well as daytime sleep time of children might be under-reported and lead to a potential risk of under-estimating the prevalence of insufficient sleep and bedtime delay. Third, this study was implemented in Songjiang District, a rural area of Shanghai; since sleeping issue in children is not only a rural topic, we need to select some urban children in future studies. Fourth, there is lack of differentiation that sleep–wake schedules and sleep duration may differ between the scheduled days and free days in kindergarten children (34). Moreover, this study also lacks objective control methods, such as the interior temperature and lightning conditions, so the incorporation of some improvements should be considered in future follow-up studies.

## Conclusion

Insufficient sleep and bedtime delay were prevalent among children aged 3 to 6 years old in Shanghai, especially in boys and older children, and TV watching as well as parents accompanying for sleep were associated with insufficient sleep and bedtime delay. We recommend that parents should limit the screen time of children, advocate earlier bedtime among children, and delay morning wake-up time among children who were awakened by their parents or alarm clock as well as make their children sleep in separate beds or rooms in younger age, which are crucial to lower the prevalence of insufficient sleep and bedtime delay in children.

## Data Availability Statement

The original contributions presented in the study are included in the article/Supplementary Material, further inquiries can be directed to the corresponding author/s.

## Ethics Statement

The studies involving human participants were reviewed and approved by Songjiang Maternal and Childcare Hospital Institution Review Board. Written informed consent to participate in this study was provided by the participant's legal guardian/next of kin.

## Author Contributions

RW and QY participated in the study design. RW and XC conducted the study and drafted the paper. YQ, QZ, RW, and QY participated in field work. BL revised the paper. All authors have read this paper and approved the final manuscript.

## Funding

This study was supported by grants from the Research Program of Shanghai Sports Bureau (20Q001), Shanghai Shenkang Hospital Development Center Management Research Program (2020SKMR-32), and the National Key R&D Program of China (2018YFC1705300). The funder had no role in the study design, data collection and analysis, decision for publication, or preparation of the manuscript.

## Conflict of Interest

The authors declare that the research was conducted in the absence of any commercial or financial relationships that could be construed as a potential conflict of interest.

## Publisher's Note

All claims expressed in this article are solely those of the authors and do not necessarily represent those of their affiliated organizations, or those of the publisher, the editors and the reviewers. Any product that may be evaluated in this article, or claim that may be made by its manufacturer, is not guaranteed or endorsed by the publisher.

## Abbreviations

TV: television
CME: Chinese Ministry of Education
CHY: Chinese Yuan
SD: standard deviation
IQR: interquartile range
OR: odds ratio
CI: confidence interval
AAP: American Association of Pediatric.
