# Supplementary material for: The Prevalence of Insufficient Sleep and Bedtime Delay Among Kindergarten Children Aged 3 to 6 Years in a Rural Area of Shanghai: A Cross-Sectional Study
Source: Front Pediatr. 2021 Nov 25;9:759318. doi: 10.3389/fped.2021.759318 (PMC8655690; doi:10.3389/fped.2021.759318)
Supplement: Supplementary file 1 [file Data_Sheet_1.PDF]

Sleeping Condition among Kindergarten Children in Shanghai

Questionnaire (translation version)

ID: □□-□□□

Part A: General Information

A1. How old is your child? _____years

A2. What is the sex of your child? (1) male (2) female

A3. Does your child have siblings? (1) yes (2) no

A4. What is the ethnics of your child?

(1) Han nationality (2) others

A5. What is your age and sex?

(1) age: _____years

(2) sex: □male □female

A6. What is your education level?

(1) 0-6 year (illiterate or primary school)

(2) 7-9 years (junior high school)

(3) 10-12 years (senior high school)

(4) 13-16 years (college)

(5) >16 years (postgraduate and above)

A7. What is your husband/wife age? _____years

A8. What is the education level of your husband/wife?

(1) 0-6 year (illiterate or primary school)

(2) 7-9 years (junior high school)

(3) 10-12 years (senior high school)

(4) 13-16 years (college)

(5) >16 years (postgraduate and above)

A9. What is the total income in your family last year?

(1) <50000 RMB

(2) 50000-10000 RMB

(3) 100001-150000 RMB

(4) 150001-300000 RMB

(5) >300000 RMB

A10. Are you a local resident? (1) yes (2) no

Part B: Sleeping Condition in Children

B1. When will your child go bed in the night usually? ___:___ (Hour:Minutes)

B2. When will your child fall asleep in the night? ___:___ (Hour:Minutes)

B3. When will your child wake up in the morning usually? ___:___ (Hour:Minutes)

B4. How many hours does your child sleep in the daytime on average? ____hours____minutes

B5. Is your child accompanied by parents for sleep in the night?

(1) always (2)sometimes (3) never

B6. How long will your child watch TV in a day on average? ____hours____minutes

B7. Does your child watch TV before sleep?

(1) yes (2) no

B8. Does your child has nightmares during night sleep?

(1) almost all the time (2)sometimes (3) never

B9. Does your child have sleep walking during night sleep?

(1) almost all the time (2)sometimes (3) never

B10. Does your child have night terrors during night sleep?

(1) almost all the time (2)sometimes (3) never

B11. Does your child grind his/her teeth in sleep?

(1) almost all the time (2)sometimes (3) never

B12. Does your child snore in night sleep?

(1) almost all the time (2)sometimes (3) never

Part C: Contact information

D1. What is your home contact phone number? □□□□□□□□

D2. What is your telephone number? □□□□□□□□□□□

D3. Phone number of investigator: □□□□□□□□□□□

D4. Investigation date: □□□□-□□-□□

Investigator signature:______________
